# Temporal regulation of planarian eye regeneration

**DOI:** 10.1002/reg2.61

**Published:** 2016-10-28

**Authors:** Michelle E. Deochand, Taylor R. Birkholz, Wendy S. Beane

**Affiliations:** ^1^ Department of Biological SciencesWestern Michigan UniversityKalamazooMI,USA

**Keywords:** Eyes, innervation, nerve degeneration, planaria, regeneration timing

## Abstract

While tissue regeneration is typically studied using standard injury models, in nature injuries vary greatly in the amount and location of tissues lost. Planarians have the unique ability to regenerate from many different injuries (including from tiny fragments with no brain), allowing us to study the effects of different injuries on regeneration timelines. We followed the timing of regeneration for one organ, the eye, after multiple injury types that involved tissue loss (single‐ and double‐eye ablation, and decapitation) in *Schmidtea mediterranea*. Our data reveal that the timing of regeneration remained constant despite changing injury parameters. Optic tissue regrowth, nerve re‐innervation, and functional recovery were similar between injury types (even when the animal was simultaneously regrowing its brain). Changes in metabolic rate (i.e., starving vs. fed regenerates) also had no effect on regeneration timelines. In addition, our data suggest there may exist a role for optic nerve degeneration following eye ablation. Our results suggest that the temporal regulation of planarian eye regeneration is tightly controlled and resistant to variations in injury type.

## Introduction

Planarians are an important model organism for the study of adult stem cell mediated tissue regeneration (Elliott & Sanchez Alvarado 2013; Rink 2013). They are amenable to manipulations such as RNA interference, and their relatively straightforward anatomy makes tissue morphology easy to track during new growth. Several species are routinely used in the laboratory, including *Schmidtea mediterranea* for which a sequenced genome exists (Robb et al. 2008, 2015). In addition, they possess a central nervous system, bi‐lobed cephalic ganglia, and a majority of the neural subtypes, neuropeptides and neurotransmitters found in vertebrates (Collins et al. 2010; Fraguas et al. 2012; Rangiah & Palakodeti 2013). Therefore, it is not surprising that planaria have become an emerging model for both nerve and eye regeneration (Emili Saló 2008; Gentile et al. 2011).

The planarians’ remarkable regenerative abilities have been studied for centuries after injuries of every conceivable type and orientation; in fact, it has been calculated that small fragments with as few as 10,000 cells (or approximately 1/279th of a whole worm) still have the ability to regenerate regardless of the tissue that is removed (Morgan 1898; Montgomery & Coward 1974). The planarian ability to be cut in many different ways, or into fragments of various sizes, and still regenerate is not found in most other regenerative model organisms. Therefore, this provides the opportunity to investigate whether it takes an individual organ the same amount of time to complete regeneration regardless of how much tissue is removed from the animal or even the nature of the tissues that are removed. For these analyses, we chose to investigate eye regeneration in planarians.

**Figure 1:**
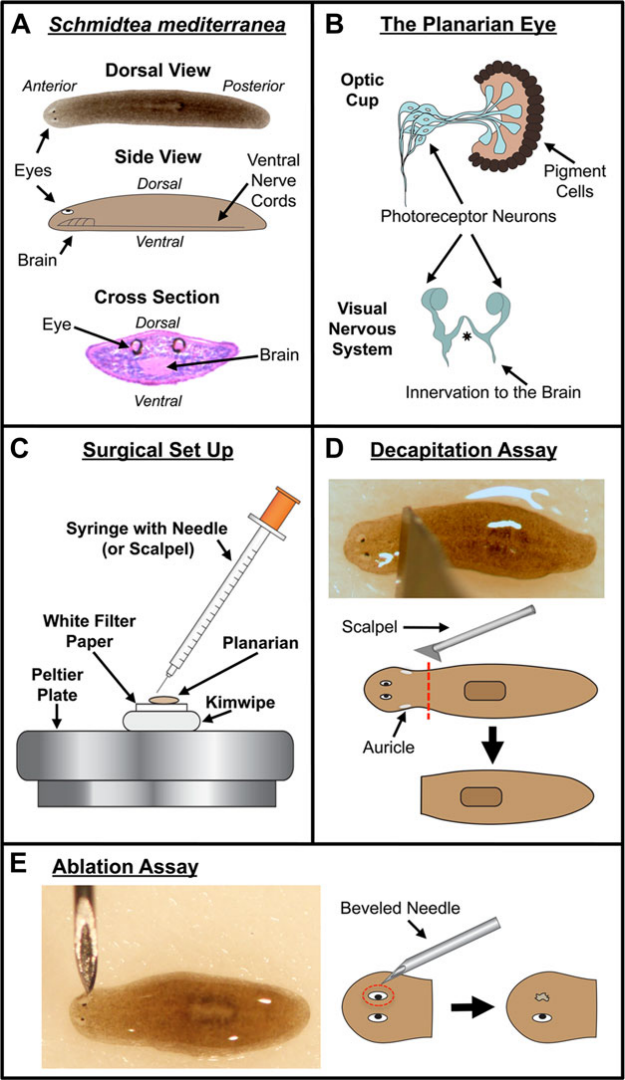
Planarian model system and microsurgical assays. (A) Planarian anatomy. Dorsal, lateral, and transverse cross‐section views. Cross‐section stained with hematoxylin and eosin (H&E). (B) Diagram of the planarian eye. Optic cup view showing the two eye tissue types, and visual nervous system view showing the optic chiasm (asterisk) and innervation to the brain. (C) Schematic view of surgery set‐up (performed under a dissecting scope). (D) Decapitation assay. Dotted line, amputation plane. (E) Ablation assay. Dotted circle, ablated region.

The planarian eye is an excellent organ for studying the temporal regulation of regeneration, as it is composed of only two tissues: photoreceptor neurons and pigment cells (Fig. 1A, B). The pigment cells form a semi‐lunar pattern on the proximal side of a transparent optic cup (Kishida 1967; Carpenter et al. 1974). The bipolar photoreceptor neurons project their dendrites into the optic cup, forming stacks of photosensitive microvilli called rhabdomeres, while their axons innervate the underlying brain both contralaterally and ipsilaterally via a true optic chiasm (Okamoto et al. 2005). Regeneration of both eye tissues is regulated by a single eye stem cell population, and planarian eye development relies on many of the same genes involved in this process in other species (Lapan & Reddien 2011, 2012). This includes opsin (rhabdomeric or r‐opsin) and other rhodopsin signaling pathway components, expressed in the light‐sensing photoreceptors (Azuma & Shinozawa 1998; Salo et al. 2002; Lapan & Reddien 2012).

Here, we address the question of whether or not the regeneration timeline of a single organ (the eye) remains constant when injury parameters are changed. We performed comparative analyses of eye regrowth over a 28‐day period in regenerates following decapitation, single‐eye ablation, or double‐eye ablation, as well as under both starved and fed conditions. Our data demonstrate that the temporal regulation of planarian eye regeneration following eye loss is not affected by modulations in the nature of the injury, the amount of tissue that is removed, or the metabolic rate of the animals. Our analyses also suggest that surgically ablated planarian optic nerves first degenerate prior to initiating photoreceptor regeneration, a finding that was unexpected. Together, these data suggest that eye organ regeneration is tightly regulated temporally in planarians and that a generalizable timeline of regenerative events can be constructed despite varying injury types.

## Results

### Type of tissue loss does not affect the timing of eye regeneration

**Figure 2:**
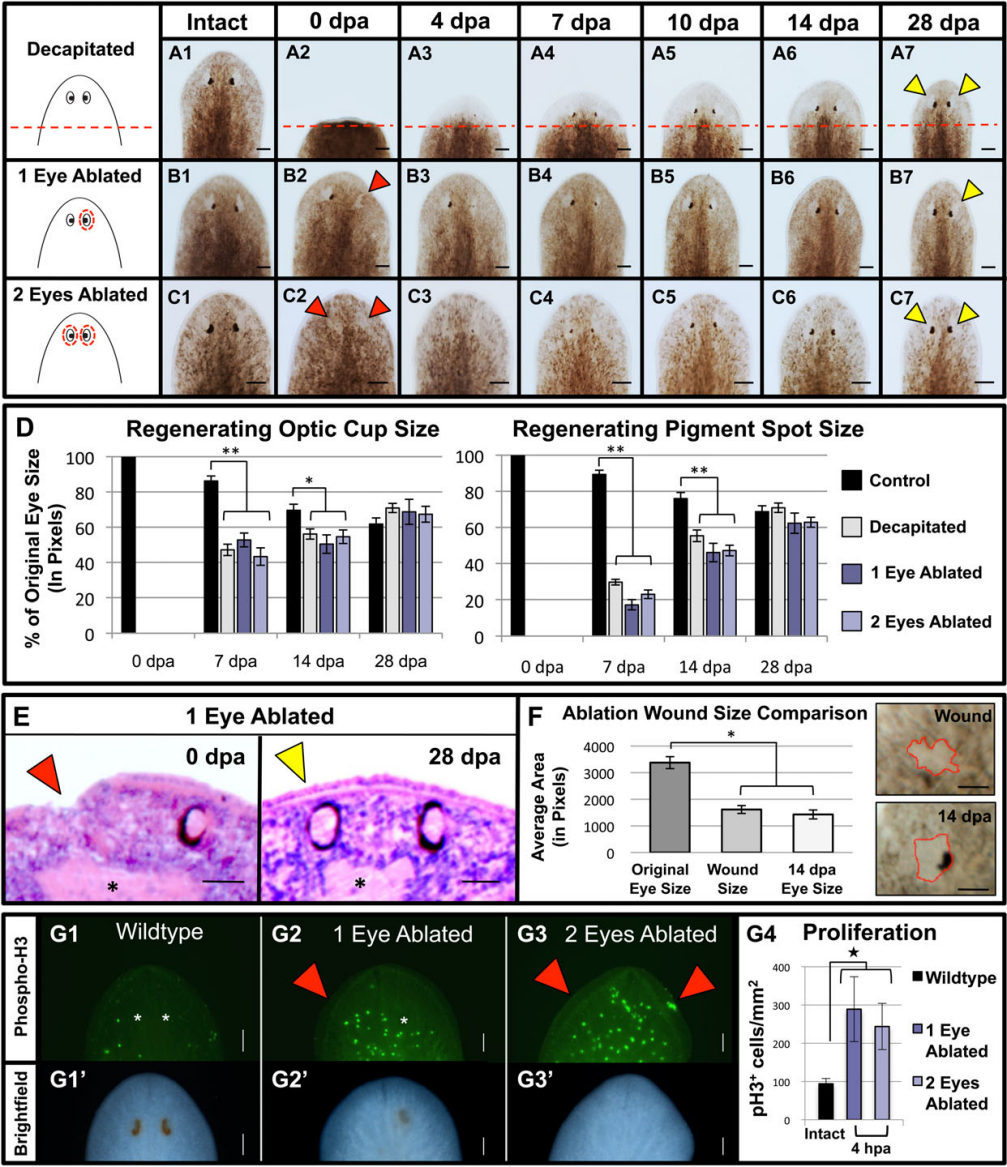
Timing of eye regeneration does not change with injury type. (A)−(C) Regeneration morphology. The same worms pictured before surgery (intact) and over 28 days of regeneration. Phenotypes shown are representative of *n* ≥ 40 in each condition. (A) Decapitated, (B) one eye ablated, and (C) both eyes ablated. Note that although eyes regenerate by 14 days in all conditions, they are smaller than intact eyes. Dotted lines, amputation planes; dotted circles, ablated regions. Scale bars: 100 μm. (D) Graph of eye sizes for the entire optic cup and the pigment spot alone compared to the original size prior to eye loss (*n* ≥ 9). **P < 0.001; *P < 0.01. (E) Cross‐sections (H&Es). Pigment cells, black tissues; Asterisks, brain. Scale bars: 25 μm. (F) Wound size (at 1 h post ablation) relative to both the eye's original size prior to ablation and the regenerated eye at day 14 (*n* ≥ 10). Insets: wound and regenerated eye in the same animal, with borders outlined in red for clarity. *P < 0.01. Scale bars: 5 μm. (G) Phospho‐histone‐H3 staining at 4 h post ablation (hpa). (G′) Brightfield images of fixed worms in (G), showing eye location/absence. Asterisks, eyes. ^*^ *P* < 0.05. Scale bars: 100 μm (*n* ≥ 4). Red arrows, ablated eyes; yellow arrows, regenerated eyes; dpa, days post amputation/ablation; error bars, standard error of the mean.

**Figure 3:**
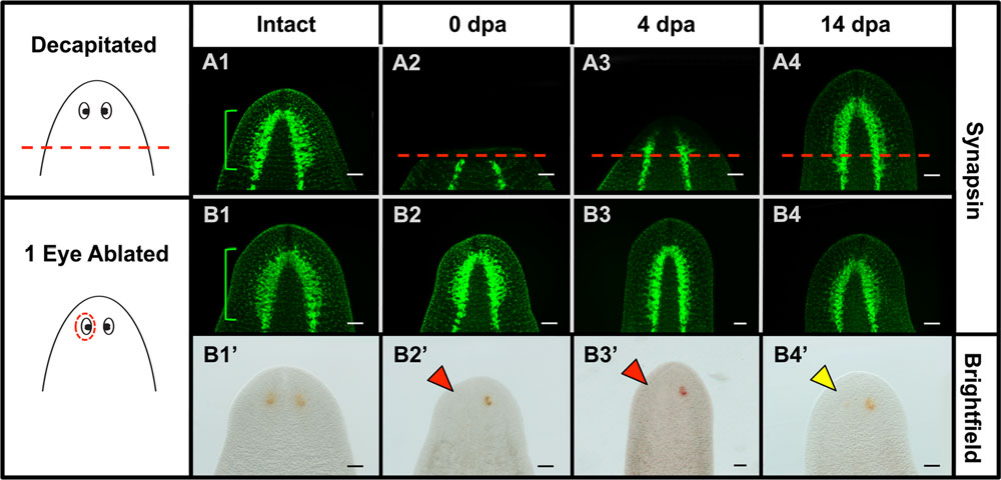
Decapitation, but not eye ablation, disturbs underlying brain structures. Anti‐synapsin immunolabeling of the nervous system. (A) Decapitated and (B) one‐eye ablated worms. (B′) Brightfield images of fixed worms in (B), shown to mark ablation site. Brain is removed following decapitation and restored by day 14, whereas the brain is undisturbed following ablation. Brackets, bi‐lobed cephalic ganglia (brain); dotted lines, amputation planes; dotted circle, ablated region; red arrows, ablated eyes; yellow arrow, regenerated eye; dpa, days post amputation/ablation. *n* = 10. Scale bars: 100 μm.

Planarian flatworms are equally able to regenerate eyes following surgical ablation of either one or both eyes (such as when the optic cup is removed using the beveled tip of a needle, Fig. 1E), as well as when the entire head is removed (such as decapitation via a transverse cut below the auricles, Fig. 1D). Surgical ablation of the eye removes mainly just the optic cup (pigment cells and the dendrites/cell bodies of the photoreceptor neurons) without significantly disturbing surrounding tissues (Figs. 2B, C and 3B). Decapitation removes not only the eyes but all anterior structures including the brain (Figs. 2A and 3A) and the anterior‐most portion of the intestinal track, requiring worm fragments to regenerate many other tissues in addition to the eyes. Furthermore, both the wound size and blastema (new tissue) produced following decapitation are larger than that following eye ablation. Therefore decapitation and ablation represent two very different injury types, involving different amounts of tissue loss and repair, in which we can study the regeneration of the same organ (the eye).

To compare the effects of these different injury types resulting in eye loss on the timing of eye regeneration, eye morphology was characterized over a 28‐day period following decapitation (Fig. 2A), single‐eye ablation (Fig. 2B), or double‐eye ablation (Fig. 2C) of *S.  mediterranea* planarians. Our data revealed that, regardless of the injury method, the timing of eye regeneration remained constant with no differences in either optic cup (eye) or pigment spot sizes (Fig. 2D). In all conditions, pigment spots were visible no earlier than day 4. By day 14, both the unpigmented optic cup and the pigment spot were regenerated; however, the regenerated eyes were all still smaller than both their original (pre‐surgery) size and uninjured controls (Fig. 2D). In single‐eye ablations, the ablated eye was also smaller than the intact, non‐ablated eye (Fig. 2B6); this size differential normalized by day 28 (Figs. 2B7 and 4A), at which point regenerates and wildtype worms became indistinguishable for all conditions. These results demonstrate that simultaneous regrowth of the brain (as part of head regeneration following decapitation, Fig. 3A) does not impact the overall time needed to complete eye regeneration. Consistent with these data, we found that the timeline for eye regeneration was also not affected when eye ablation was paired with simultaneous tail amputation (*n* = 40, data not shown). Together, these data suggest that the temporal regulation of eye regeneration is not affected by the size or location of the tissues removed.

**Figure 4:**
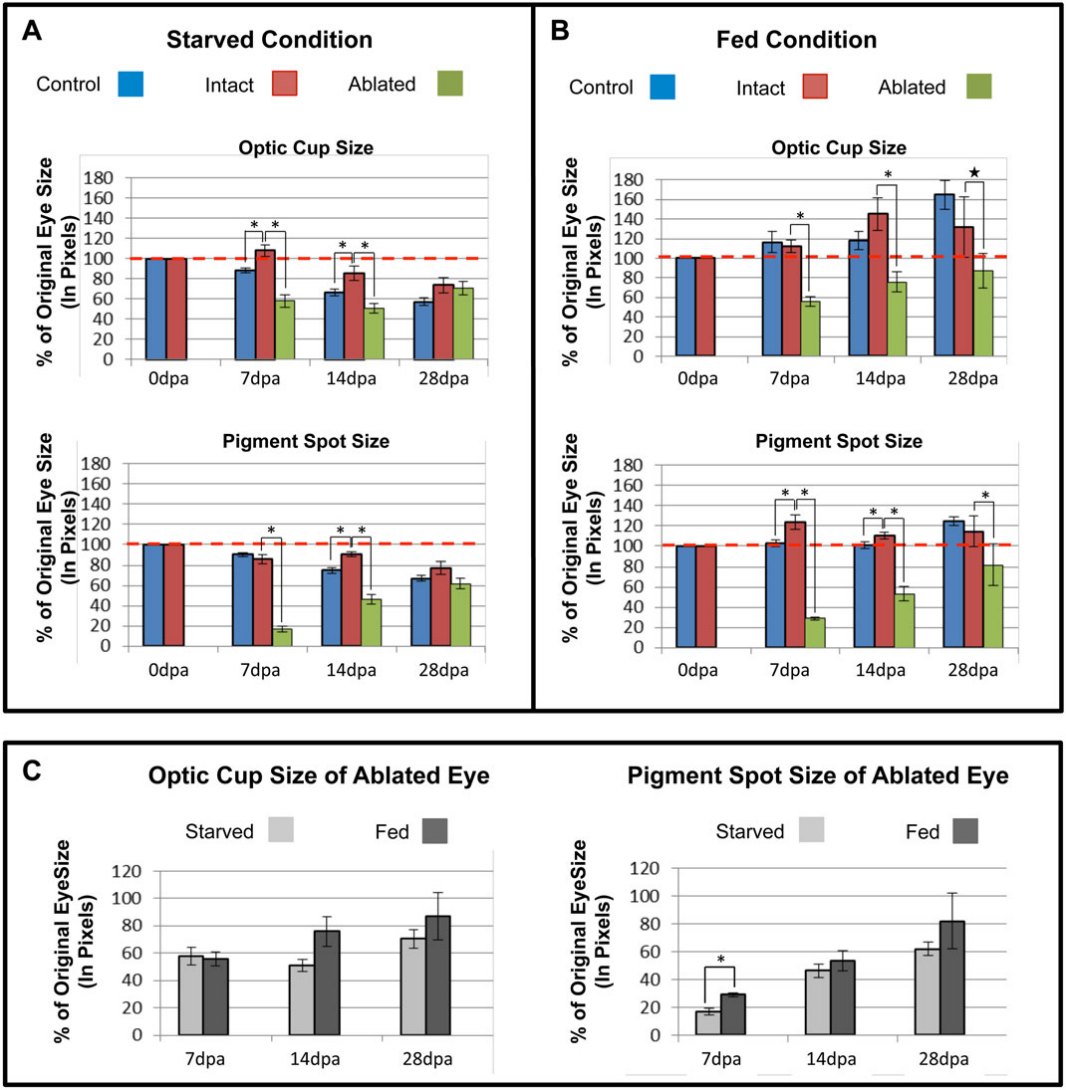
Timing of eye regeneration does not change with food intake. (A) Starving worms, *n* ≥ 10. (B) Feeding worms, *n* ≥ 9. For both conditions, one eye was ablated and regeneration in the same worm was followed for 28 days. Both overall optic cup size (top graphs) and pigment spot size (bottom graphs) were measured, as a percentage of the original eye size (size of the eye prior to its ablation). Control, eyes in non‐ablated worms; intact, non‐ablated eye from ablated worms; ablated, ablated eye from ablated worms; dashed line, control level. (C) Comparison of ablated eyes only. Graphs of just the ablated eye data from (A) and (B). Final 28‐day average optic cup sizes in starved (70.39%) and fed (86.95%) worms were not statistically significant (*P* = 0.4), nor were average pigment spot sizes (starved 61.92% and fed 81.83%, *P* = 0.36). Dpa, days post ablation. ^*^ *P* ≤ 0.01; ^*^ *P* = 0.02. Error bars, standard error of the mean.

Our finding that surgical ablation of a single eye resulted in a size difference between the ablated and unablated eye at day 14 was unexpected, as 2 weeks is the standard time point at which the completion of regeneration is typically scored in planarians. One possible reason for this size difference could be that too much tissue underneath the optic cup was removed during ablations. Examination of histological cross‐sections at 1 h post ablation showed that our ablation technique does not disturb the underlying brain or the remaining non‐ablated intact eye (Fig. 2E). This was confirmed by synapsin labeling of the central nervous system, which showed that surgical eye ablation does not disrupt overall brain architecture (Fig. 3B). Together, these data reveal that tissue removal during ablation was largely restricted to the optic cup. The data suggest that completion of eye regeneration (or at least the allometric scaling of new eye tissues) in planarians takes between 14 and 28 days.

We observed that, following wound constriction in ablated worms, a smaller area (than the original eye) was produced to which regenerating eye tissues initially appeared to be confined. Analyses of wound sizes at 1 h post ablation revealed that the wound site contracted to an average of 47.83% of the original eye size prior to ablation and this size was not statistically different from the overall eye size later on day 14 (*P* = 0.42, Fig. 2F). This might suggest that eye regeneration occurs via morphallaxis (rearrangement/reallocation of existing cells) rather than epimorphosis (new tissue growth) (Agata et al. 2007). In planarians, following decapitation there occurs a peak of stem cell proliferation around 4 h post amputation (prior to blastema outgrowth) that is characteristic of epimorphic regeneration (Wenemoser & Reddien 2010). Therefore, we analyzed mitoses following eye ablation using anti‐phospho‐histone‐H3 staining, which specifically labels the actively dividing planarian stem cell population (Fig. 2G). Our data demonstrated that at 4 h both single‐eye and double‐eye ablation resulted in an upregulation of proliferation (Fig. 2G4). Furthermore, our data revealed that after single‐eye ablation the proliferative response was concentrated specifically on the ablated (rather than the non‐ablated) side (Fig. 2G2), while after double‐eye ablation the proliferative response was spread across the entire anterior region—including near the anterior pole, where dividing cells are not normally found (compare Fig. 2G3 to 2G1). Together, these data suggest that in planarians ablation of the optic cup results in the formation of new eye tissues via proliferation.

### Metabolic rate does not affect the timing of eye regeneration

Planarians are typically not fed during regeneration assays, because following food intake planarian stem cells undergo a burst of proliferation that first peaks around 6 h and lasts 24 h to several days post feeding, depending on species (Baguna 1974; Gonzalez‐Estevez et al. 2012; Tu et al. 2012). However, starving planarians become smaller due to degrowth—a process of metabolizing pre‐existing tissues while at the same time proportionally re‐scaling overall worm size (Baguna 1974; Oviedo et al. 2003; Gonzalez‐Estevez et al. 2012). Thus, we wanted to investigate whether degrowth may have contributed to the normalization of ablated eye size at 28 days (since the overall worm size was shrinking). Additionally, we wanted to investigate whether differences in metabolic rate (such as after feeding) affect the timing of eye regeneration. Therefore, following surgical ablation of a single eye, regeneration was quantified over 28 days in worms that were fed once a week, compared to starving worms (Fig. 4). The area of the optic cup (the white unpigmented region composed of photoreceptors, plus the black pigment cells) and the area of the pigment spot alone were measured. For both starved (Fig. 4A) and fed (Fig. 4B) conditions, eye sizes were analyzed in non‐ablated control worms (blue bars), the intact eye of ablated worms (red bars), and the regenerating ablated eye of the same ablated worms (green bars). These data confirmed that, as expected, both optic cup and pigment spot sizes in control worms decreased when animals were starving but increased when worms were fed (Fig. 4A, B, blue bars).

Interestingly, in both starved and fed conditions, intact pigment spot size at day 14 was statistically larger than control pigment spot size in uninjured worms, and this was also true on day 7 for feeding worms (Fig. 4A, B). Similarly, in starving worms at days 7 and 14 the overall optic cup size of intact eyes was statistically larger than that of control eyes (Fig. 4A). These size differences, between the intact (untouched) eye in animals undergoing ablation of the other eye and the eyes of control (uninjured) animals, normalized by day 28 for all conditions. These findings were unexpected and the reasons for the differences are unclear, but they may indicate the possibility that regenerative processes interact or interfere in some manner with normal growth/degrowth processes.

Consistent with our morphological observations (Fig. 2), in starving worms the optic cup and pigment spot sizes of the ablated eye were statistically smaller than that of the intact eye at day 14, but were equivalent at day 28 (Fig. 4A). These data show that in degrowing animals eye size normalizes within 1 month. However, when animals were fed once a week (Fig. 4B), optic cup and pigment spot sizes between ablated and intact eyes remained unequal at day 28. These data suggest that at day 28 the unequal eye size in fed worms is probably due to the increased size of the intact eye, while similarly the equal eye size in starved worms is due to the decreased size of the intact eye. Importantly, direct comparison of just the ablated eyes in starved versus fed worms (Fig. 4C) demonstrates that there is no difference in the actual size of either the regenerated optic cup or pigment spot regardless of feeding at either day 14 or day 28 (Fig. 4C, *P* ≥ 0.36). These data demonstrate that while nutritional intake (metabolic rate) does affect the overall growth or degrowth of the worm, it has no effect on the rate of eye regeneration.

### The timing of functional eye recovery is not affected by injury type

**Figure 5:**
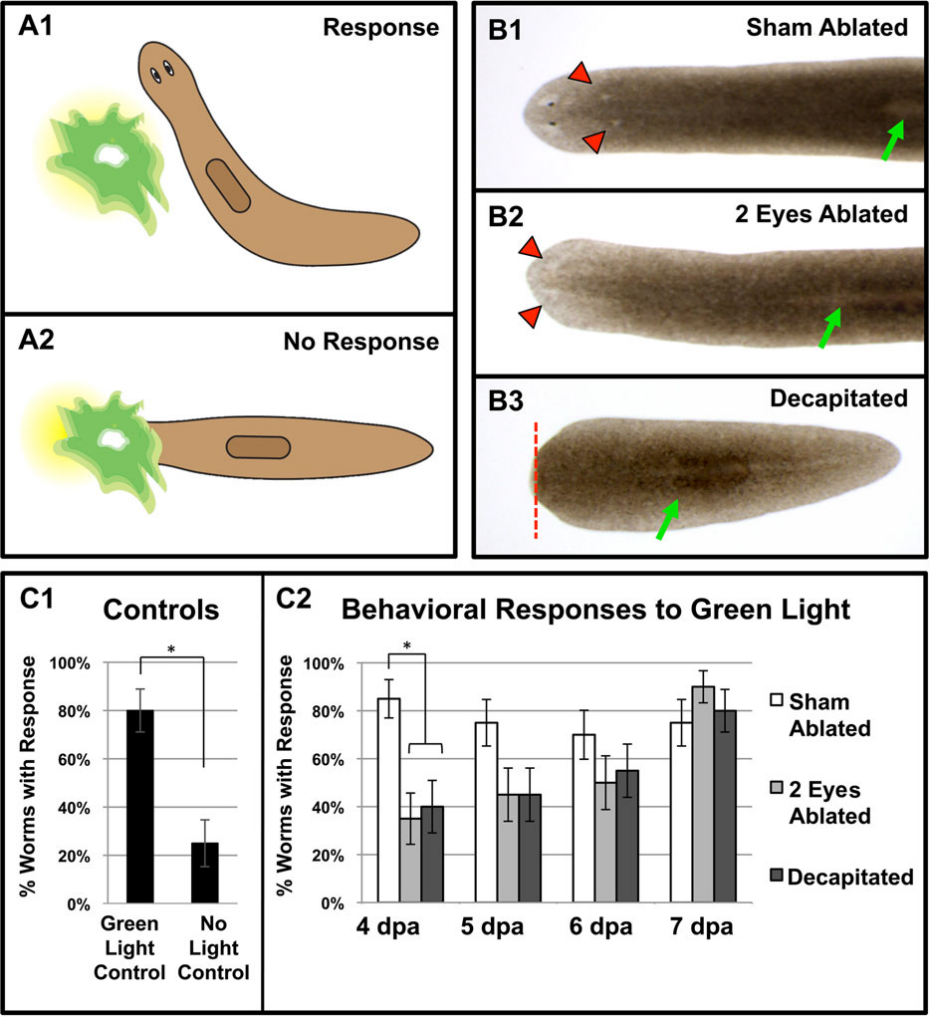
Timing of functional eye recovery does not change with injury type. (A) Behavioral phototactic assay. Photophobic planarians avoid traveling through green wavelengths. Examples of (A1) a positive behavioral response, where worms turn away from the light, and (A2) no response, where worms travel directly through the light. (B) Injury types. (B1) Sham ablated, (B2) both eyes ablated, and (B3) decapitated worms at 24 h post injury. Red arrowheads, ablation sites; dotted line, amputation plane; green arrows, pharynx. (C) Behavioral responses. (C1) Positive (green light) and negative (no light) controls using uninjured wildtype worms. Without any light cues, 25% of worms randomly change direction (“response”). (C2) Responses over time. On day 4 of regeneration, both decapitated and double‐eye ablated worms fail to respond to green light (not significantly different from no light controls, *P* ≥ 0.32). Photophobia reappears starting on day 5, with all worms functionally recovered by day 7. Dpa, days post amputation/ablation. ^*^ *P <* 0.01. Error bars, standard error of proportion. *n* = 20.

Our morphological analyses indicate that injury type does not change the timing of when eye tissues regenerate. However, this does not necessarily correspond to equivalent functional recovery times, especially since worms needing to regrow their brain as well may recover function slower than worms just regrowing an optic cup. To determine the timeline of when the regenerating eye first becomes functional, we analyzed regenerates using a light avoidance assay we previously developed (Paskin et al. 2014) (Fig. 5). Planarians have a well‐established photophobic response and will seek cover when exposed to light. Their phototactic responses vary by wavelength, with green wavelengths causing a moderate behavioral response where worms turn to avoid traveling through the light (Paskin et al. 2014). Functional recovery of visual phototransduction was tested starting at 4 days post ablation (when pigment spots were first visible, Fig. 2), and then on each subsequent day in the same worms through day 7.

Worms were assayed as they moved towards a pinpoint of light (composed of green wavelengths) and scored for the presence of a photophobic response (where the worm avoided the light) or the absence of a response (where the worm traveled through any part of the light) (Fig. 5A). We tested and compared the responses of sham ablated regenerates (who received two similar‐sized wounds just posterior to the eyes but still in the head region), double‐eye ablated regenerates, and decapitated regenerates (Fig. 5B). Because worms change direction randomly while traveling, “no‐light” controls were performed where the light cue was never presented (Fig. 5C1). These data show that the background “response” level for this assay is 25%, in which worms deviated from their initial trajectory and were scored as having a “response” (Fig. 5C1). For all days (4−7 post injury), the sham ablated surgery controls displayed a photophobic response (Fig. 5C2) similar to that of uninjured wildtype worms (*P* = 0.68, Fig. 5C1). In contrast, both the decapitated and double ablated regenerates failed to respond at day 4 (Fig. 5C2), with response levels that were not statistically different from the no‐light controls. Over the next 3 days, the number of decapitated and double‐eye ablated regenerates that displayed a response increased, with approximately half of the worms avoiding light on days 5 and 6, until on day 7 there was no significant difference in the responses between sham, double‐eye ablated, and decapitated animals (Fig. 5C2). These data suggest that the functional recovery of regenerating eyes does not begin until after day 4 and is completed by day 7.

**Figure 6:**
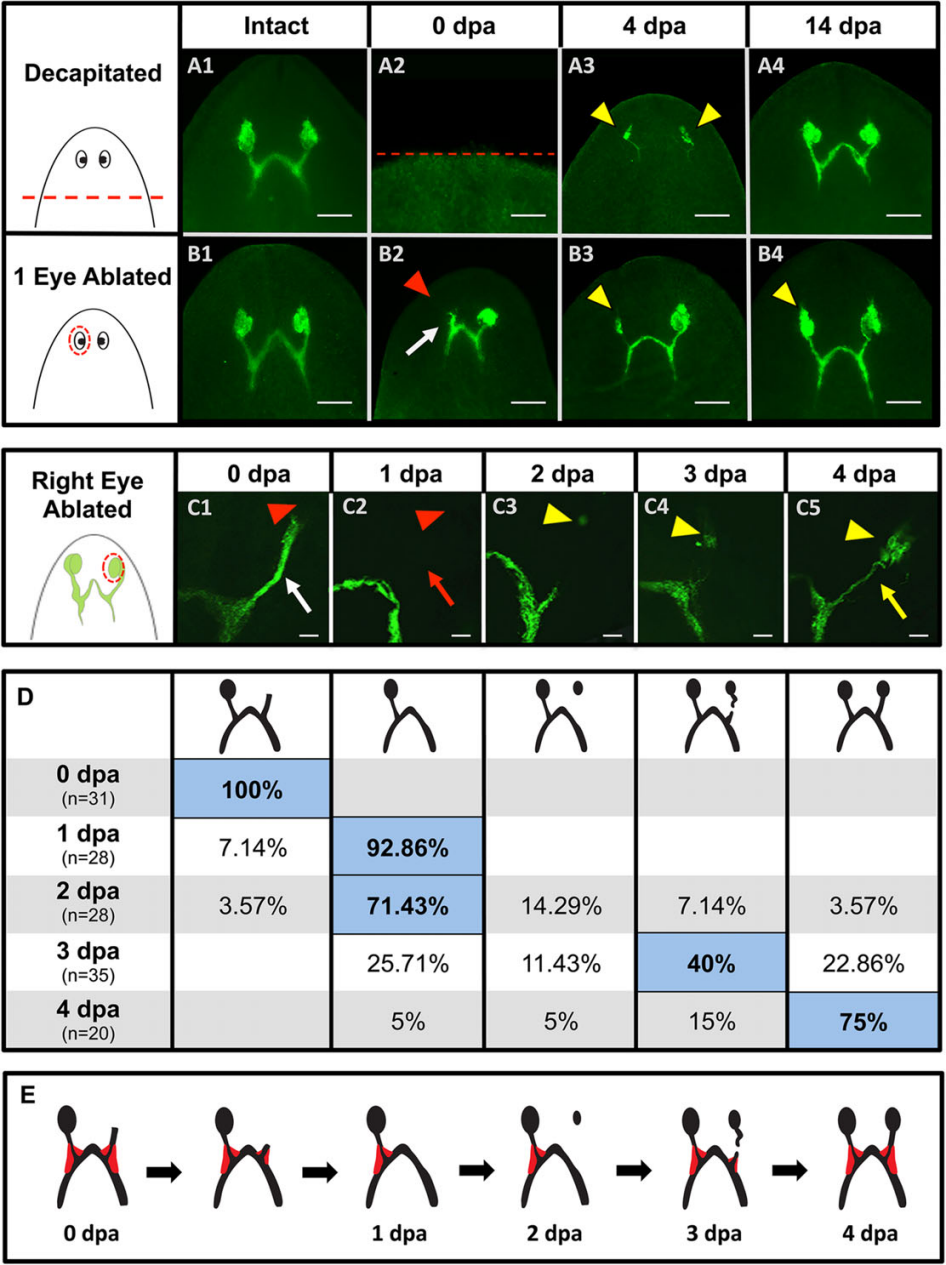
Timing of optic nerve regeneration does not change with injury type. (A)−(C) Anti‐arrestin immunolabeling. (A) Decapitated and (B) one‐eye ablated worms both initiate photoreceptor regeneration by day 4, and complete regeneration by day 14. Dotted lines, amputation planes. *n* = 10. Scale bars: 100 μm. (C)−(E) Optic nerve re‐innervation following ablation. (C) Confocal *z*‐stacks. At 1 h post ablation the original innervation (C1, white arrow) from the ablated optic cup is still visible, but is gone by day 1 (C2, red arrow). Photoreceptors in the new optic cup reappear by day 2 (C3). Processes extend from both optic cup and chiasm by day 3 (C4), finally meeting on day 4 (C5, yellow arrow). Scale bars: 15 μm. (D) Optic nerve regeneration phenotypes by percentage. Majority phenotype in blue highlight. (E) Diagram of the neural “bulge” (in red). The neural bulge is the last part of the optic nerve to be lost. Red arrowheads, ablated eyes; yellow arrowheads, regenerating eyes.

These functional data also suggest that, regardless of the injury type, optic nerve re‐innervation is not complete prior to day 4. Using a marker of planarian photoreceptor cells (anti‐arrestin), we examined the timing of optic nerve regeneration following both decapitation and surgical ablation of the optic cup. Our data reveal that at 4 days post injury photoreceptor cell bodies and optic nerve processes have regenerated in both cases—with both injuries showing complete restoration of optic nerve sizes by day 14 (Fig. 6A, B). These data are consistent with our behavioral response data, showing that functional recovery begins after day 4. Together, the data suggest that differences in injury type or size have no effect on either the timing of functional recovery during planarian eye regeneration or the temporal regulation of optic nerve re‐innervation following eye loss.

### Following optic cup ablation optic nerves degenerate prior to regenerating

Unexpectedly, our initial analyses of optic nerve regeneration revealed that after surgical ablation a neural “stalk” remained attached to the optic chiasm (white arrow in Fig. 6B2), which then seemed to disappear during new photoreceptor cell body regeneration (Fig. 6B3). Therefore, we more closely examined this process by looking at the recovery of optic nerves every 24 h following ablation (Fig. 6C−E). The data show that removal of the optic cup does in fact leave a stalk of neural processes connected to the optic chiasm on the ablated side, a stalk which is still present immediately following ablation (white arrow in Fig. 6C1). By 24 h post ablation, this stalk is lost—along with the ipsilateral neural “bulge” where the optical neurons meet in the optic chiasm (Fig. 6C2, E). Only after this degeneration (around 48 h) do photoreceptor cell bodies begin to regenerate at the site of the new optic cup (but without any processes extending towards the optic chiasm) (Fig. 6C3). By day 3, the photoreceptor cells begin to extend processes towards the optic chiasm, while simultaneously processes also extend upwards from the optic chiasm towards the newly regenerated photoreceptors, and the neural “bulge” reappears (Fig. 6C4, E). By day 4 post ablation, the majority of these processes have met and now connect (Fig. 6C5, D). These data are consistent with our previous data, suggesting that innervation is not complete and eye function not restored prior to day 4.

**Figure 7:**
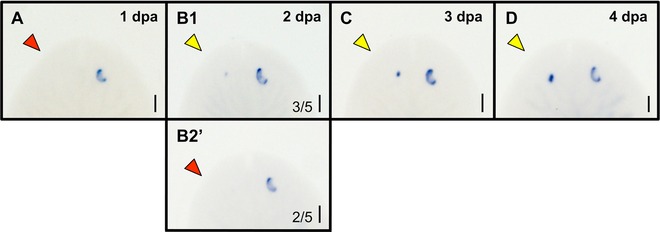
The timing of pigment cell regeneration parallels the timing of optic nerve regeneration. (A)−(D) Tyrosinase labeling of pigment cells over time. Whole mount in situ hybridizations in one‐eye ablated worms (left eye) through day 4. Normal tyrosinase expression is revealed by the intact (right) eye. Note that pigment cell regeneration does not start until day 2, when photoreceptor cells also begin regenerating. *n* ≥ 5. Phenotypic penetrance is 100%, except on day 2 (B1, B2). Dpa, days post ablation; red arrows, ablated eye; yellow arrows, regenerating eye. Scale bars: 100 μm.

**Figure 8:**
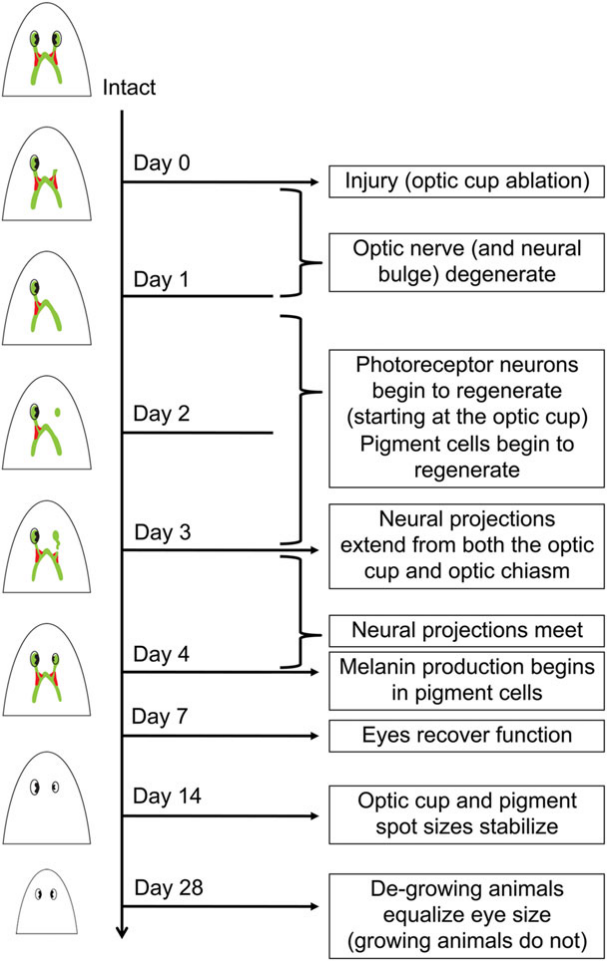
Model of eye regeneration timeline, with surgical ablation as example injury. Green, visual nervous system; red, ipsi/epsilateral neural bulge; black crescent, pigment cells.

By morphological observation, the pigmented spot of the planarian eye was not visible until day 4, well after our data show that photoreceptor regeneration has begun. To more closely examine the timeline of pigment cell regeneration in relation to that of photoreceptor regeneration, we analyzed eye regeneration every 24 h post single‐eye ablation using a molecular marker of pigment cells (Fig. 7). The marker tyrosinase is part of the melanin synthesis pathway and specifically labels pigment cells in planarians (Lapan & Reddien 2011). Our data reveal that, similar to photoreceptor neuron regeneration, pigment cell regeneration begins on day 2 after optic nerve degeneration has occurred (Fig. 7B), concurrent with the onset of photoreceptor regeneration. These data suggest that, following eye loss, photoreceptor and pigment cell regeneration occur in parallel, while melanin production by pigment cells begins after the photoreceptor neurons reconnect with the optic chiasm on day 4. Together, our data reveal that the coordinated series of events during planarian eye regeneration are under tight temporal regulation (Fig. 8).

## Discussion

Regeneration timelines vary by both species and the organ that is regenerated. Invertebrate head regeneration in *Hydra* is complete in 3 days, whereas in planaria it takes about 2 weeks (Beane et al. 2013; Vriz et al. 2014). Vertebrate appendage regeneration of fins in zebrafish takes 3 weeks to complete, but regeneration of the tail in *Xenopus* (including the spinal cord) takes only 1 week, while the regeneration of salamander limbs takes several months, depending on the species (Tsonis 1996; Tseng & Levin 2008; Pfefferli & Jaźwińska 2015). This complicates our ability to extrapolate information about the temporal regulation of tissue regeneration from one animal model to another. However, understanding the temporal regulation of organ regeneration will be critical for formulating novel repair strategies, as injuries rarely occur in nature with the same precision of both method and location that occurs in the laboratory setting. Thus studies are needed to determine whether or not injury parameters affect the timing of regenerative events, and if so which parameters have effects on regenerative timelines and in which organs and species.

Our studies demonstrate that following eye loss there is a set timeline of events that occurs in planarians (Fig. 8), which is distinct from the degree of injury or nutritional input (metabolic rate). This includes differences in blastema size (blastemas formed after ablation are smaller than after decapitation) or the amount or identity of extra tissues needing to be regenerated (for instance, surgical ablation requires regeneration of mostly just the optic tissues, whereas decapitation also requires regeneration of all anterior tissues including the brain). Despite variations in both metabolic rate and injury type, eye regeneration still took 2 weeks to complete. During the first 4 days, both optic nerve and pigment cell regeneration occurred—culminating with melanin production by pigment cells starting on day 4. Following this, recovery of eye function was completed in the next 3 days (within 1 week post injury), while optic cup size was stabilized by day 14. The timing of optic nerve regeneration coincides with previous studies (Inoue et al. 2004); however, neural regrowth from projections at both the optic chiasm and the optic cup has yet to be described for planarian eye regeneration.

In vertebrates, peripheral nervous system axons when cut undergo an active, regulated process known as Wallerian degeneration prior to regeneration (Fang & Bonini 2012; Conforti et al. 2014; Freeman 2014). This process disintegrates the remaining axon distal to the injury site. Our immunohistological analyses suggest that, similar to Wallerian degeneration, planarian optic nerves initially degenerate during the first 24 h after ablation of the optic cup and before the onset of photoreceptor regeneration (Fig. 6). The disappearance of the remaining “stalk” of photoreceptor processes (which project dorsally towards the missing optic cup), and more importantly the ipsilateral neural “bulge” (formed where the ipsilateral and contralateral nerves cross in the optic chiasm), strongly suggests that the optic nerve may degenerate rather than simply contract from neural bifurcation. The fact that photoreceptor regeneration does not begin until after this process is complete implies that neural degeneration may be an important component of optic nerve regrowth in the context of regeneration of the optic cup. Further studies will be required to determine if degeneration is in fact required for subsequent regeneration to occur, as seen with zebrafish peripheral sensory axons (Martin et al. 2010).

Previous studies of planarian eye regeneration following decapitation describe growth cones of optic neurons growing out from the regenerating photoreceptor cells before extending down into the brain via a new optic chiasm (Inoue et al. 2004; Yamamoto & Agata 2011), which we also observed (Fig. 6A3). However, our data reveal that when only the optic cup is removed the neural response also includes optic nerve growth upward from the optic chiasm. It is not known whether the regenerating photoreceptor cells attract the partially degenerated nerve in the optic chiasm (or vice versa), although our observations were that processes were always seen simultaneously extending from both the new photoreceptor cells and the optic chiasm (prior to reconnection) (Fig. 6C4). Classical studies of crustacean neural regeneration, as well as data from urodeles, *Caenorhabditis elegans*, and leech (among other species), describe regeneration via the process of axonal fusion, where regenerating neurons stretch towards each other from both sides (Hoy et al. 1967; Macagno et al. 1985; Neumann et al. 2011; Zukor et al. 2011). Conversely, new axonal projection post decapitation (which occurs in one direction solely from the photoreceptors to the brain) is more representative of both planarian embryonic eye development (Martin‐Duran et al. 2012) and eye development in other species, including vertebrates (Mann et al. 2004). These data suggest that the planarian optic nerve provides an excellent *in vivo* model system in which to elucidate the mechanisms that regulate photoreceptor neuroregeneration, innervation, and patterning.

It has previously been shown that a single adult stem cell precursor population gives rise to both planarian eye tissue types during regeneration: the photoreceptor neurons and the pigment cells (Lapan & Reddien 2011, 2012). In agreement with this model, our data show that the onset of photoreceptor and pigment cell regeneration both occur on day 2 (Figs. 6 and 7), after optic nerve degeneration is completed. These data suggest that both eye tissue types are regenerated simultaneously following eye loss. Interestingly, while loss of the photoreceptor neurons (via RNAi to OtxA) does not appear to affect pigment cell regeneration, loss of pigment cells (via RNAi to either Sp6‐9 or Dlx) does result in defective axonal patterning of the photoreceptors (Lapan & Reddien 2011). These data suggest that pigment cells may be required for maintenance of optic chiasm patterning and point to a delicate relationship between the optic neurons and pigment cells during both regeneration and homeostasis.

Our data suggest that the temporal regulation of eye regeneration, and by extension the eye stem cell population, is surprisingly resistant to certain changes in environment (such as amount of tissue loss, injury type, and metabolic rate). One important future direction will be to screen for genes that can alter the regeneration timeline, either increasing or decreasing temporal control of eye regrowth. In particular, identifying genes that can increase the speed of organ regeneration (without compromising organ structure or function) would be of interest for repair strategies. Identifying pathways that regulate the timing of eye regeneration would also increase our understanding of the temporal regulation of stem cell differentiation during organ regeneration.

## Materials and methods

### Colony care

The CIW4 clonal line of asexual *Schmidtea mediterranea* was used and maintained at 19°C as previously described (Beane et al. 2012), except that worm water was composed of 0.5 g/L of Instant Ocean. Worms 4−8 mm in length were starved for ≥1 week prior to use, except where noted (feeding assay).

### Microsurgeries

Worms were immobilized via chilling on a custom Peltier plate covered with a moist Kimwipe topped by white filter paper (Whatman #2, which improves visibility of eye spots) (Fig. 1C). Worms were decapitated with a scalpel just below the auricles (Fig. 1D). Removal of either one or both eyes was performed by scooping the eye out with the beveled tip of a 31‐gauge insulin needle (5/16” in length), where the syringe was used as a handle (Fig. 1C, E). Following surgery, worms were placed into fresh worm water and allowed to regenerate at 20°C in the dark. Sham surgeries were performed as above except that tissue was removed posterior to the eyes (but still in the head region). These wounds approximated the same amount of tissue as removed for double‐eye ablations and resulted in the formation of blastemas.

### Feeding assay

Worms 4−8 mm in length were starved for ≥1 week, and then either the right or left eye was surgically ablated. Individual worms were kept in non‐treated welled plates and worm water was changed twice a week. Worms were fed the day following ablation, and then once a week for 1 month, with liver paste dyed with food coloring to confirm consumption.

### Immunofluorescence

Worms were fixed in Carnoy's and processed as in Beane et al. (2012). Primary antibodies: anti‐arrestin, 1:10,000 (kind gift from M. Levin, Tufts University, Medford, MA); anti‐synapsin, 1:50 (Developmental Studies Hybridoma Bank 3C11, Iowa City, IA), and anti‐phospho‐histone‐H3 (Sigma/Millipore 04‐817, St. Louis, MO), 1:25. Secondary antibodies: for arrestin and synapsin, goat anti‐mouse Alexa 488, 1:400 (Molecular Probes A‐11001, Eugene, OR); for H3P, goat anti‐rabbit horseradish peroxidase (Invitrogen 65‐6120, Carlsbad, CA) with TSA Cy3‐Tyramide amplification (Perkin Elmer, 1:50, Waltham, MA).

### *In situ* hybridization

Whole‐mount *in situ* hybridization was performed based on the protocol described in Pearson et al. (2009) but with the following modifications. For fixation, 7.5% *N*‐acetyl cysteine was used, the reduction solution step was omitted, and worms were stored in MeOH. For bleaching, samples were rehydrated into Phosphate Buffered Saline with Triton X (PBSTx), rinsed in 1× saline sodium citrate (SSC), then placed in formamide bleaching solution overnight as described in King & Newmark (2013). Prior to post‐fix, samples were rinsed in 1× SSC, then twice in PBSTx, followed by a 10 μg/mL proteinase K treatment. For NBT (nitro blue tetrazolium)/BCIP (5‐bromo‐4‐chloro‐3‐indolyl‐phosphate) developing, Sigma FAST tablet (B‐5655) was dissolved in 7.3 mL of alkaline phosphatase (AP) buffer with 10% polyvinyl alcohol plus 2.7 mL nuclease‐free water. *Smed‐tyrosinase* (Lapan & Reddien 2011) probe was used at 1 ng/μL. Anti‐digoxigenin‐AP (Roche, Basel, Switzerland) was used, 1:3000.

### Histology

Worms were fixed using Carnoy's, stored in EtOH at −20°C, and processed as previously described (Stevenson & Beane 2010). Worms were embedded in paraffin wax and cut into 7‐μm sections with a microtome (Leitz 1512, Leica, Wetzlar, Germany). Slides were removed of wax and rehydrated with a series of washes in xylene and alcohol, respectively. Tissues were stained with hematoxylin and eosin (Fisher, Waltham, MA) and mounted with Permount (Fisher) following standard protocols.

### Functional analyses

An avoidance assay was performed as previously described (Paskin et al. 2014), using a commercially available green laser pointer with a nominal peak wavelength of 532 nm (± 10 nm). The same group of sham ablated, double‐eye ablated, and decapitated worms were assayed in the dark at 4, 5, 6, and 7 days post ablation, when worms were moving in a straight line towards the light source (positioned about one diameter of the circle of light, or ∼2.5 mm, in front of the worm). Positive controls (wildtype, uninjured worms) and “no‐light” negative controls (where wildtype worm trajectory was scored for 2.53 sec without the light cue being presented) were also performed. Each worm was tested twice for a total of 20 trials for each condition.

### Image collection

Unless otherwise noted, all images were collected using a Zeiss V20 fluorescence stereomicroscope with AxioCam MRc or MRm camera and Zen Lite software (Zeiss, Oberkochen, Germany). The following were exceptions: Figure 1(D), (E) was taken with a Canon EOS Rebel T5i SLR camera (Canon, Tokyo, Japan) mounted on a dissection scope, while anti‐arrestin confocal stacks were taken on a Nikon Eclipse Ti Confocal microscope with NIS‐Elements software (Nikon, Tokyo, Japan). Adobe Photoshop (Adobe Systems, San Jose, CA) was used to orient and scale images and improve clarity (not for anti‐synapsin or anti‐arrestin photos). Data were neither added nor subtracted; original images available upon request.

### Quantitative and statistical analyses

Optic cup measurements (defined as the white unpigmented region plus the pigmented region) and pigment spot measurements (pigmented region only) were quantified by using the area (number of pixels) at each time point, expressed as a percentage of that eye's area prior to ablation. For all eye (and wound) size measurements images were taken at the same magnification and resolution, and significance was determined by Student's *t*‐test. For functional analyses (avoidance assay) significance was determined using a two‐sample *t*‐test between percentages. *P* ≤ 0.01 was significant (except where noted).
